# Development and validation of a clinical prediction model for patient-reported pain and function after primary total knee replacement surgery

**DOI:** 10.1038/s41598-018-21714-1

**Published:** 2018-02-21

**Authors:** M. T. Sanchez-Santos, C. Garriga, A. Judge, R. N. Batra, A. J. Price, A. D. Liddle, M. K. Javaid, C. Cooper, D. W. Murray, N. K. Arden

**Affiliations:** 1 Musculoskeletal Epidemiology, Nuffield Department of Orthopaedics, Rheumatology and Musculoskeletal Sciences, University of Oxford, Oxford, UK; 2 MRC Lifecourse Epidemiology Unit, University of Southampton, Southampton General Hospital, Southampton, UK; 3 Arthritis research UK Centre for Sport, Exercise and Osteoarthritis, Nuffield Department of Orthopaedics, Rheumatology and Musculoskeletal Sciences, University of Oxford, Oxford, UK

## Abstract

To develop and validate a clinical prediction model of patient-reported pain and function after undergoing total knee replacement (TKR). We used data of 1,649 patients from the Knee Arthroplasty Trial who received primary TKR across 34 centres in the UK. The external validation included 595 patients from Southampton University Hospital, and Nuffield Orthopaedic Centre (Oxford). The outcome was the Oxford Knee Score (OKS) 12-month after TKR. Pre-operative predictors including patient characteristics and clinical factors were considered. Bootstrap backward linear regression analysis was used. Low pre-operative OKS, living in poor areas, high body mass index, and patient-reported anxiety or depression were associated with worse outcome. The clinical factors associated with worse outcome were worse pre-operative physical status, presence of other conditions affecting mobility and previous knee arthroscopy. Presence of fixed flexion deformity and an absent or damaged pre-operative anterior cruciate ligament (compared with intact) were associated with better outcome. Discrimination and calibration statistics were satisfactory. External validation predicted 21.1% of the variance of outcome. This is the first clinical prediction model for predicting self-reported pain and function 12 months after TKR to be externally validated. It will help to inform to patients regarding expectations of the outcome after knee replacement surgery.

## Introduction

Total knee replacement (TKR) surgery is a common procedure with 86,438 performed in 2014 in England, Wales, Northern Ireland and the Isle of Man^1^; and 7,169 primary knee arthroplasties performed in 2013 in Scotland^2^. It is one of the most effective surgical interventions, where patients experience substantial improvements in mental health, satisfaction and functional status in addition to a decrease in pain, and these effects are maintained over the long term^3^. However, up to 20% of patients are not satisfied with their outcome^4–6^.

Patient-reported outcome measures (PROMs) are typically recorded before and after surgery for TKR, using tools such as the Oxford Knee Score (OKS) which measures pain and functional status^7^. Pain and functional status after knee surgery depends on a wide range of factors, such as age^8,9^, gender^10,11^, socioeconomic status^12,13^, social support^14^, mental health^14,15^, pain and function before the surgery^16^, number of comorbidities^17^, and also implant and hospital type^18^. Although these studies provide information on different risk factors, they have been unable to explain much of the variability in outcome following surgery.

In clinical medicine, a multivariable prediction model combines information from multiple predictors to predict the probability of or risk for a specific disease or outcome^19^, with the purpose of informing patients and guiding clinicians in decision-making on further health service strategies.

Therefore, as an example application of clinical prediction model, we developed and externally validated a simple prediction model for improvement in pain and function 12 months after TKR using data from the Knee Arthroplasty Trial (KAT)^20,21^ (development dataset) and the Clinical Outcomes in Arthroplasty study (COASt) (validation dataset).

## Results

### Descriptive statistics

In the development cohort (KAT), we analysed information on 1,649 patients who agreed to complete both pre- and post-operative OKS questionnaires. We used information on 595 patients in the validated cohort (COASt). Patient characteristics from derivation and validation studies (case mix) are shown in Table 1. Broadly, patient’s characteristics were generally similar in the development and the validation studies. Compared with the COASt study, patients in the KAT study were more likely to present anxiety or depression and damaged pre-operative anterior cruciate ligament (ACL), they also had lower ASA grading system and were less likely to have other condition affecting mobility and pre-operative fixed flexion deformity.

**Table 1:** Patient characteristics in the development and the validation cohorts. KAT, knee arthroplasty trial; COASt, clinical outcomes in arthroplasty study; OKS, Oxford knee score; IQR, interquartile range; sd, standard deviation; BMI, body mass index; EQ-5D-3L, 3-level version of the EuroQol five dimensions questionnaire; IMD, index of multiple deprivation; ASA, physical status classification system of the American society of anaesthesiologists; ACL, anterior cruciate ligament.

Variable	KAT (1649)		COASt (n = 595)	
	n		n	
**OKS at follow-up (units**, **median**, **IQR)**	1,649	36 (27–42)	595	39 (30–44)
**Age at operation (years**, **%)**				
<*60*	168	(10.2)	65	(10.9)
*60–69*	613	(37.2)	206	(34.6)
*70–79*	691	(41.9)	246	(41.3)
*80 or more*	177	(10.7)	78	(13.1)
**Gender (%)**				
*Male*	728	(44.2)	256	(43.0)
*Female*	921	(55.8)	339	(57.0)
**Marital status (%)**				
*Married*	1,082	(66.0)	330	(67.1)
*Single*	65	(4.0)	20	(4.1)
*Widowed/divorced*	492	(30.0)	142	(28.9)
**IMD 2004 score (median**, **IQR)**	1,026	15.6 (9.6–25.5)	594	10.2 (6.3–16.9)
**BMI** (**mean**, **sd)**	1,597	29.7 (5.4)	595	30.7 (5.5)
**EQ**-**5D-3L 5th question (%)**				
*Anxiety/depression*	643	(39.3)	173	(32.3)
*No*	993	(60.7)	362	(67.7)
**OKS baseline (units**, **mean**, **sd)**	1,649	18.3 (7.5)	542	19.4 (7.7)
**ASA grade (%)**				
*Fit and healthy*	277	(17.5)	49	(9.4)
*Asymptomatic no restriction*	991	(62.8)	381	(73.4)
*Symptomatic minimal/severe restriction*	311	(19.7)	89	(17.2)
**Disease type (%)**				
*Osteoarthritis*	1,561	(95.3)	465	(94.5)
*Rheumatoid arthritis*	77	(4.7)	27	(5.5)
**Disease side (%)**				
*One knee*	432	(26.4)	83	(20.3)
*Both knees*	642	(39.2)	192	(46.8)
*General*	564	(34.4)	135	(32.9)
**Previous knee arthroscopy (%)**				
*No*	1,420	(86.7)	472	(89.4)
*Yes*	218	(13.3)	56	(10.6)
**Other condition affecting mobility (%)**				
*No*	1,411	(86.3)	56	(10.3)
*Yes*	224	(13.7)	487	(89.7)
**Fixed flexion deformity (%)**				
*No*	690	(42.6)	151	(37.3)
*Yes*	930	(57.4)	254	(62.7)
**Pre-operative ACL (%)**				
*Intact (*<*5* *mm)*	1,054	(65.4)	282	(75.2)
*Damaged (*≥*5* *mm)*, *absent*	558	(34.6)	93	(24.8)

Distribution of outcome and potential predictors in responders and non-responders in the development and the validation cohorts are presented in Supplementary Table S1. Responders in the KAT tended to have better pre-operative OKS, better physical status, and less anxiety or depression scores than non-responders. Responders in the COASt study were less often singles, had less anxiety or depression and were less likely to present general disease (see Supplementary Table S1).

#### Missing data

Missing values for all variables included in the analysis are given in Supplementary Table S2. For the majority of factors in the development and the validation datasets the proportion of missing data was ≤20%. However, missingness was higher for socioeconomic status in the KAT dataset and for disease side, fixed flexion deformity and pre-operative ACL in the COASt dataset.

### Predictors of Outcome – model development

Of the 14 variables entered into a backward regression model, ten variables were identified as predictors of post-operative OKS in the KAT study. Regression coefficients and 95% confidence intervals (CI) for each predictor are shown in Table 2. A positive regression coefficient value indicates that the group had better post-operative pain/function and a negative value indicates that the group had worse post-operative pain/function.

**Table 2:** General linear model identifying predictors of knee pain and function 12-month after total knee replacement in the development dataset. OKS, Oxford knee score; IMD, index of multiple deprivation; BMI, body mass index; ASA grade, physical status scoring according to the American society of anaesthesiologists; ACL, anterior cruciate ligament; EQ-5D-3L, 3-level version of the EuroQol five dimensions questionnaire; CI, confidence interval. Variables included in the final regression model are those that are retained in at least 70% of the 200 bootstrap backward selection regression models.

Intercept and Predictors (reference category)	Overall (n = 1,649)		
	% Retained in final model	Coefficient (95% CI)	*P*-value
**Intercept**		32.9	
**Age at operation** (<60 years)			
*60–69 years*	100% (forced)	0.8 (−1.5 to 3.1)	0.477
*70 to 79 years*	100% (forced)	1.4 (−0.9 to 3.6)	0.238
*80 years or more*	100% (forced)	−2.5 (−5.5 to 0.4)	0.088
**Gender** (Female)			
*Male*	100% (forced)	−4.8 (−8.0 to −1.6)	0.003
**Age x gender** (<60 years x female)			
*60–69 years x male*	90%	4.8 (1.4 to 8.3)	0.006
*70 to 79 years x male*	84%	4.3 (0.8 to 7.7)	0.015
*80 years or more x male*	100%	8.1 (4.0 to 12.3)	<0.001
**IMD 2004 score (10 units)**	97%	−0.6 (−1.0 to −0.2)	0.008
**BMI**, **kg/m**^**2**^ **(10 units)**	96%	−1.5 (−2.4 to −0.6)	0.001
**EQ**-**5D-3L 5th question** (No)			
*Anxiety/depression*	98%	−1.6 (−2.5 to −0.6)	0.001
**OKS baseline**	100%	0.4 (0.3 to 0.4)	<0.001
**ASA grade** (Fit and healthy)			
*Asymptomatic no restriction*	55%	─	─
*Symptomatic minimal/severe restriction*	97%	−2.0 (−3.2 to −0.8)	0.001
**Previous knee arthroscopy** (No)			
*Yes*	82%	−1.6 (−3.0 to −0.2)	0.025
**Other condition affecting mobility** (No)			
*Yes*	100%	−3.3 (−4.7 to −2.0)	<0.001
**Fixed flexion deformity** (No)			
*Yes*	100%	1.7 (0.8 to 2.6)	<0.001
**Pre-operative ACL** (Intact)			
*Damaged/absent*	81%	1.0 (0.1 to 2.0)	0.029
**R** ^**2**^			**19**.**3%**
**Optimism**			**1**.**6**
**Bias-corrected R** ^**2**^			**17**.**6%**

Worse pre-operative OKS, self-reported anxiety/depression, presence of ASA grade 3/4 (compared to fit and healthy), presence of other conditions affecting mobility and previous knee arthroscopy were strongly associated with worse outcome. Increasing deprivation score and increasing BMI were associated with decreasing OKS at 12 months follow-up (worse pain/function). Presence of fixed flexion deformity and damaged/absent ACL (compared with intact) were significantly associated with better outcome at 12 months after TKR.

A significant interaction between age and gender was found (*P*-value < 0.001) and included into the final model. Patients aged younger than 60 and older than 80 presented a worse pain and functional status at 12 months after knee surgery, and this effect also varied by gender. Younger women (age < 60) had better outcome than men; but in the oldest age group (age 80 or more) women had worse outcomes than men. There was no difference of gender on OKS outcome in the middle age groups (age 60 to 80).

### Internal validation

**Figure 1:**
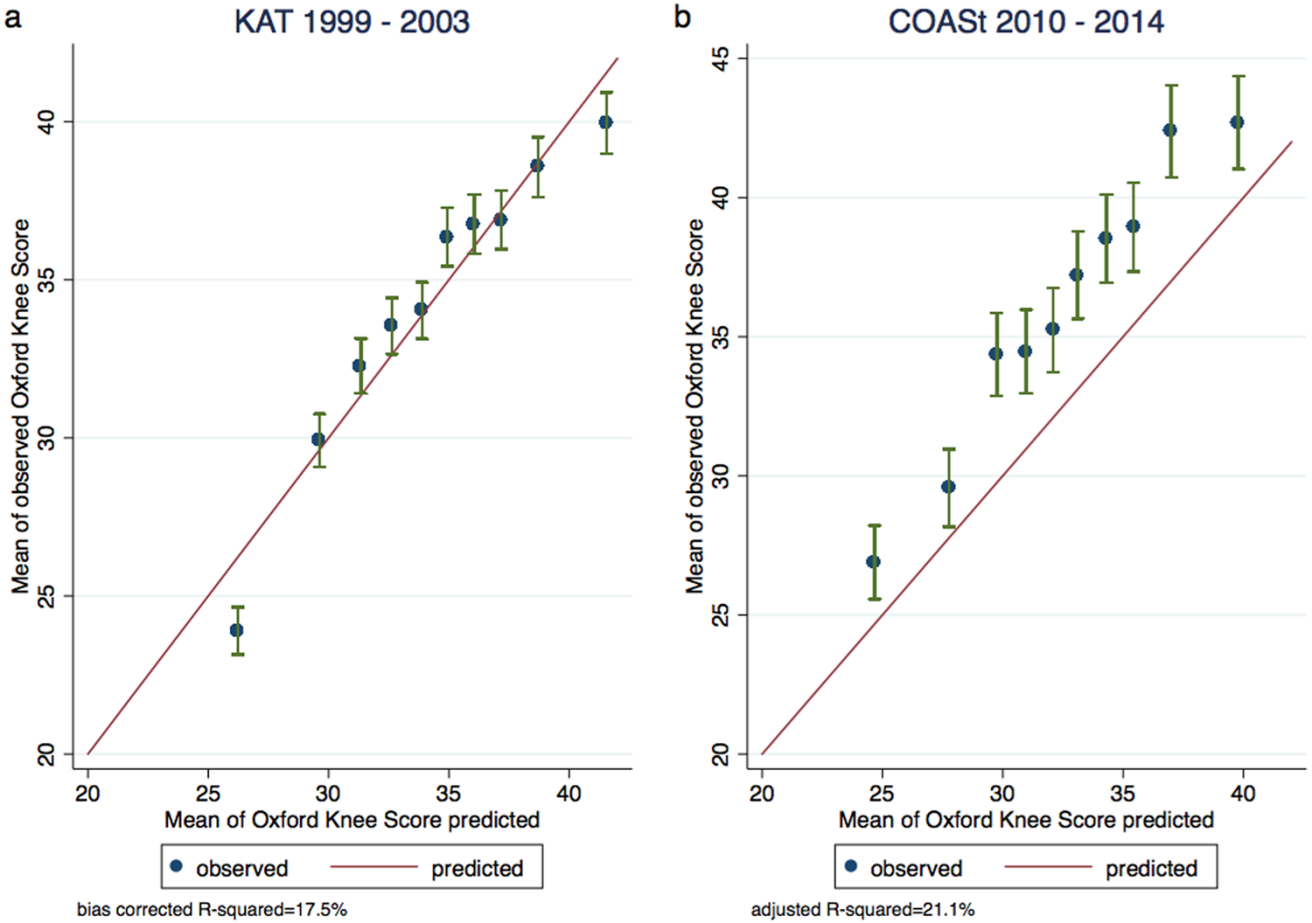
Calibration plot with R^2^. (**a**) Calibration plot of the imputed development dataset (n = 1,649). (**b**) Calibration plot of the external dataset also imputed (n = 595). Internal and external samples used for validation were divided in 10 deciles, according to their predicted risk. For each decile, the mean predicted risk and the mean observed cases are shown on the X and Y axes, respectively. Bars indicate 95% Poisson confidence intervals.

The bias-corrected R^2^ statistic in the final predictive model including the patient and clinical factors was 17.6%. Age, sex and pre-operative OKS explained 12.8% of the variability in outcome, when the other patients’ characteristics were included 14.7% of the variance of outcome was explained, reaching 17.6% when clinical variables were added. Model calibration was good, with close agreement between predicted and observed values of post-operative OKS at 12 months after TKR (Fig. 1).

### External validation

The model showed better discriminatory ability than the model internally validated with an adjusted R^2^ of 21.1%. Calibration shows underestimation of the predicted values with regard to the observed data in COASt but with close agreement between both scores (Fig. 1).

## Discussion

We have developed and externally validated a new prediction model for patient-reported pain and function after TKR by using patient characteristics and clinical variables that are easy to measure. To our knowledge, this is the first study to attempt external validation for predicting outcome after TKR. The model showed adequate predictive validity with an R^2^ of 17.5% and had good calibration across all deciles of predicted 12-month OKS. The external validation improved prediction up to 21.1% but this prediction underestimated observed OKS.

Presence of a fixed flexion deformity, and an absent or damaged pre-operative ACL (compared with intact ACL) were all significantly associated with better outcome.

Determinants of worse outcome included in the final model were: worse pre-operative OKS, living in poor areas, high BMI, worse mental health, worse ASA grade, presence of other condition affecting mobility and knee arthroscopy.

All predictors have undergone internal validation using bootstrap techniques to ensure they were consistently identified as significant predictors.

This prediction model provides an individualised estimate of post-operative OKS, and change in OKS, and this information will help to inform to patients regarding expectations of the outcome after knee replacement surgery.

Few papers have described clinical risk prediction models for outcomes of knee arthroplasty^13,22–24^, however these studies are informative for decision-making but require confirmation and external validation in new patients cohorts.

We included known risk factors in our model and report coefficients similar in both magnitude and direction to those reported elsewhere for outcome after replacement. Within our study, we found that worse pre-operative knee pain and function score and greater socioeconomic deprivation, based on residential area, were significantly associated with worse patient reported outcomes, and these associations have been well documented^12–14,16,25,26^. We also found that patients with self-reported anxiety/depression were most likely to have worse post-operative OKS. These results have been consistent with previous reports, using measures of mental health such as the SF-36^27^ and the EQ-5D-3L^13^. Within this study higher BMI was associated with worse post-operative OKS. There is controversy with respect to BMI, previous studies found association between high BMI and worse outcome^28,29^ whereas others found no evidence of an association^13,30^. A possible explanation for this association may be that as BMI is known to be associated with limited physical performance^31^, the judgment of obese patients on their health status may be based on functional mobility. Although the relationship was statistically significant, the effect size was not a clinically important, meaning that BMI should not be a barrier to surgery.

In relation to the clinical variables, we found that patients with worse pre-operative physical status (ASA grade 3–4), compared to fit and healthy, pre-operative disability and previous knee arthroscopy were associated with worse outcome, and these results were consistent with other studies^3,18^.

We found that patients with a fixed flexion deformity, or an absent pre-operative ACL, achieved better outcomes one year after TKR, even after adjustment for pre-operative OKS and this finding has been previously reported in other study using KAT data^32^. Patients with a pre-operative fixed flexion deformity were more likely to present absent/damaged pre-operative ACL in this study. Also, these patients presented with similar or even better pre-operative characteristics compared with those without fixed flexion deformity and intact pre-operative (data not shown).

This study has several strengths: (1) Large sample size. A total of 1,649 patients were included in the develop analysis (2) the use of a wide range of predictor variables, including socio-demographic and almost unique data on clinical factors, (3) the use of multiple imputation and bootstrapping as an internal validation technique^33^, to ensure significant predictors are and not anomalous to this dataset, (4) in addition good reproducibility of the model has been confirmed by external validation^34,35^, and (5) collating these risk factors together to develop a clinical prediction model that may be informative for decision making.

There are some potential limitations to this study. First, although calibration of the prediction model was good for predicting attained post-operative OKS, and change (improvement in OKS), it does not currently predict dichotomous “good” or “poor” outcome (e.g. a change in score of less than 5 points”). This model could be tested against such definitions once they have been agreed.

Second, potential predictive factors, principally radiographic variables (e.g. Kellgren and Lawrence (KL) grade of OA), were not available in this study. In addition, lifestyle measures such as smoking, alcohol consumption and exercise were not available in the development dataset, and hence we did not consider them in this study. We therefore used BMI and area deprivation, which have been shown to correlate well with behavioural risk factors^36^. Similarly, race was not collected in the KAT study, and hence this variable was not included in the analysis. Since patients included in the COASt study were predominantly white (over 95%), and both studies had similar patient’s characteristics, the results of this study may not be generalizable to other racial groups. Future research should focus on predictors of outcome after TKR in different racial groups.

Third, nonresponse bias from this study limits the generalizability of study findings. Nonresponses were more likely to have worse outcome and reported anxiety and depression, thus this implies that the true effects of these predictors may be underestimated in this study. Importantly, external validation has confirmed the calibration and discrimination ability of the model.

Finally, R^2^ values were relatively low, predictive factors explained 17.5% and 21% of the variability in outcome, suggesting that the nature of the health status in TKR patients is multifactorial. However, since strong statistically significant predictors were found, we can still draw important conclusions about how changes in the predictor values are associated with changes in the outcome. Those values are consistent with other studies attempting to explain the variability in outcome of TKR^13,22,23^ and also with other well-known prediction tools such as QRISK and Framingham score that explain around 30% of the variability in outcome^37^.

We have developed and for the first time externally validated a clinical prediction model for outcome 12-month after TKR. Clinicians could use information on the level of patient outcome improvement, when counselling patients about the prognostic of TKR, allowing to the patients to be involved in the decision whether to undergo surgery. External validation has confirmed its performance and validity and it can be already used in clinical practice.

## Patients and Methods

### Data sources

#### Development dataset

This study was carried out using data from the KAT trial^20,21^: a pragmatic, partial-factorial, unblinded randomised controlled trial (International Standard Randomized Trial No. ISRCTN45837371). Patients were recruited from July 1999 to January 2003 through a random sample, stratified by surgeon according to age group, gender and site of disease. The KAT study contains information on patients receiving primary TKR across 34 centres in the UK.

#### Validation dataset

COASt, is a prospective, longitudinal cohort including patients waiting for hip and knee operations across two UK tertiary hospitals: Southampton University Hospital, and Nuffield Orthopaedic Centre (NOC) in Oxford. Southampton University Hospital provides services to some 1.3 million people, whereas NOC serves a population of around 655,000 people. Patients were recruited between 2010 and 2014.

### Participants

#### Development dataset

Patients were eligible for inclusion in KAT if a decision had been made for them to undergo primary TKR^21^. A participant was not eligible if the surgeon considered a particular type of operation to be clearly indicated (metal-backed tibial component, patellar resurfacing and/or a mobile bearing). 4,070 potentially eligible patients were found and 2,374 (58%) provided their consent and were randomised. Of those, 22 were later discarded because were randomised in error, which left 2,352 participants in the trial. For the purpose of the present study, 1,649 individuals (41%) with available data about OKS at baseline and year 1 were selected.

#### Validation dataset

Patients included had osteoarthritis (OA) or rheumatoid arthritis (RA). They were over 18 years and were competent and willing to consent to undergo primary TKR. 1,674 patients who underwent knee replacement were initially accepted by COASt study. Fifty-seven patients (3%) were excluded because of (a) duplication, (b) ineligibility, (c) withdrawn, (d) limited consent, (e) preoperatively assessed knee was different than the actual operated knee, or (f) any data provided. Patients undergoing patella-femoral resurfacing (n = 16, 1%), TKR revision (n = 112, 7%) and unicompartmental knee replacement (n = 643, 38%) were also excluded. Finally, 595 answering about OKS at year 1 (36%) were used for the external validation.

### Outcome

The outcome was the patient’s pain and functional status as measured by the OKS at 12-month after primary TKR. OKS is a validated patient-administered questionnaire which consists of 12 questions relating to knee pain and physical function limitations during the past 4 weeks^38^. Each question is on a Likert scale taking values from 0 to 4, with 4 being the best outcome. A total score was created ranging from 0 (severe symptoms and dysfunction) to 48 (no problem on any item)^7^. OKS questionnaires were also completed by participants pre-operatively. Patients were required to answer OKS questionnaires thinking of their operated knee in development and validation studies.

### Predictor variables

The *pre-operative patient characteristics* included were age, gender, marital status, socioeconomic deprivation measured by Index of Multiple Deprivation 2004 (IMD)^39^, BMI and mental health (Table 3). As the association of age on outcome was non-linear, we considered age according to the categories used in the randomization process: less than 60 years; 60 to 69 years; 70–79 years and 80 years or older. The IMD 2004, based on patients’ residential postcodes, combines weighted scores for each postcode in seven deprivation domains, where a high score indicates increased deprivation. BMI was calculated at baseline as the ratio of the weight to the square of height in meters (kg/m^2^). Values of IMD 2004 and BMI were collapsed each 10 units. Mental health was assessed using the anxiety/depression item of the European Quality of life-five domain (EQ-5D-3L) questionnaire^40^.

**Table 3:** List of prognostic variables available for analysis.

Variable	Additional information
***Patients’ characteristics***	
Age (years)	Less than 60 = 1; 60–69 = 2; 70–79 = 3; 80 or older = 4
Gender	Male = 1; female = 0
Marital status	Married = 1; Single = 2; Widowed/divorced = 3
Index of Multiple deprivation 2004	Score range from 2.1 to 79.3 (more deprived); 10 units
Body Mass Index (BMI)	Weight (kg)/height^2^ (meters); 10 units
Anxiety/depression (EQ-5D-3L 5^th^ question)	Moderate or severe = 1; None = 0
Oxford knee score (OKS) baseline	0 (worst) to 48 (best); right knee
***Clinical factors***	
Pre-operative American Society of Anesthesiologists (ASA) physical function score	Grade 1 = 1, fit and healthy; grade 2 = 2, mild disease; grades 3 and 4 = 3, incapacitating disease and life-threatening disease (grades 3 and 4 were collapsed)
Disease type	Osteoarthritis = 1; rheumatoid arthritis = 0
Disease side (Charnley classification)	One knee = 1; Both knees = 2; General = 3
Knee arthroscopy	Yes = 1; No = 0
Other condition than osteoarthritis or rheumatology arthritis affecting mobility	Yes = 1; No = 0 Lung problems (e.g. asthma), heart conditions (e.g. angina, heart attack), high cholesterol, hypertension, inflammatory joint, back pain, gout, sciatica, osteoporosis, avascular necrosis, polymyositis, polymyalgia, osteomyelitis, psoriatic arthropathy, multiple sclerosis, fibromyalgia, cerebral palsy, Parkinson’s disease, Raynaud’s syndrome, Paget disease, or childhood conditions
Fixed flexion deformity	Yes (knee that is unable to fully extend to 0) = 1; No = 0
State of anterior cruciate ligament (ACL)	Intact (<5 mm) = 0; Damaged (≥5 mm) or Absent = 1

The following variables were considered as potential *clinical risk factors*: (a) physical status such as is classified by the American Society of Anesthesiologists (ASA) grade. This grading system is a standard assessment of the patient’s general physical health prior to surgery^41^. It is composed by four categories (1, fit and healthy; 2, mild disease; 3, incapacitating disease; and 4, life-threatening disease) but for this study the last two categories were collapsed, (b) disease type (OA, RA), (c) disease side (left, right)^42^, (d) previous knee arthroscopy (it happened in any time for the development dataset while for the validation dataset it only referred to the 12 previous months before TKR), (e) presence of other conditions affecting mobility, (f) pre-operative fixed flexion deformity and (g) pre-operative status of anterior cruciate ligaments (ACL) (Table 3).

Clinical pre-operative factors refer to the operated knee in the development and the validation datasets.

### Statistics

All analyses were conducted using the Stata version 13.1 statistical software (StataCorp, College Station, Texas). To determine selection (response) bias, an analysis was conducted to compare patient characteristics in responders and non-responders to both pre-operative and 12 months post-operative OKS questionnaires in the development dataset.

General linear models were used to identify predictors on post-operative OKS. Linearity of continuous variables with the outcome was assessed using fractional polynomials and collinearity between variables was assessed by the variance inflation factor (VIF). Because the variance of the residuals is non-constant (evidence of heteroscedasticity), robust standard errors were used with the sandwich variance estimator^43^. Interactions between age and sex with all other factors were tested. To get the fit of the final model with the smallest number of variables and the best predictive validity, we followed these steps^44–46^: Step 1: multiple imputed dataset using chained equations was generated to investigate the impact of missing data^47^. Forty imputed datasets were generated using all potential factors (including the outcome) and estimated parameters were combined using Rubin’s rules; Step 2: bootstrap linear regression model method with the sandwich variance estimator was used. We drew 200 bootstrap samples with replacement from the combined 40 imputed datasets. Within each bootstrap sample, automatic backward selection was applied using a significance level equal to 0.157 (except age and gender were force-entered into all models)^48^; Step 3: variables which appeared at least 70% of the time were retained in the final regression model.

#### Internal validation

To check the internal validity of the model, 200 bootstrap samples with replacement combined with multiple imputations was once again used to assess bias-corrected estimates of predictive ability^43^. Predictive ability was assessed by examining measures of discrimination (R^2^ statistic) and calibration^48,49^. Samples were divided in ten deciles for calibration according to their predicted risk. For each decile, means of predicted and observed OKS were obtained. For each observed mean was also calculated its 95% Poisson confidence interval.

#### External validation

For the external validation, the missing values of the predictors selected in the prediction model were also imputed using the same procedure describe above. We applied the pooled coefficients of the model developed in KAT with the forty imputed datasets of COASt, and we then calculate discrimination and calibration. Discrimination was calculated by the pooled R^2^ statistics using Fisher’s r to z transformation^50^.

#### Sensitivity analysis

Finally, to evaluate whether there were differences in post-operative OKS for patients with RA versus OA, we conducted an analysis excluding patients with RA; because no substantial model differences were observed, these supporting analysis are not shown.

### Ethics

For the KAT trial ethical approval was obtained from by the Multi Centre Research Ethics Committee for Scotland in November 1998 (research protocol MREC/98/0/100) and was approved by the Local Research Ethics Committees in each study centre recruiting trial participants.

COASt has been approved by the Oxford REC A (Ethics Reference: 10/H0604/91). The sponsoring organisation of the study is the University Hospitals Southampton NHS Foundation Trust (UHS).

Both cohorts confirm to national guidelines and individual ethics and data protection requirements. Data were collected within the two cohorts in an anonymised format as confirmed by the study participants in their written informed consent and as directed by the ICH-GCP guidelines and appropriate local and International legislation. It was not be possible to re-identify the donors. Datasets were stored at the University of Oxford in a secure database. The data storage, management and handling were protected in accordance with European Commission Directive 95/46/EC and appropriate national regulations.

## Electronic supplementary material

Supplementary Tables S1 and S2
