# Supplementary material for: Development and validation of a clinical prediction model for patient-reported pain and function after primary total knee replacement surgery
Source: Sci Rep. 2018 Feb 21;8:3381. doi: 10.1038/s41598-018-21714-1 (PMC5821875; doi:10.1038/s41598-018-21714-1)
Supplement: Supplementary file 1 — Supplementary Tables S1 and S2 [file 41598_2018_21714_MOESM1_ESM.doc]

**MANUSCRIPT TITLE:**

Development and validation of a clinical prediction model for patient-reported pain and function after primary total knee replacement surgery

**Author names:**

MT Sanchez-Santos^1,3, ¶^, C Garriga^1, ¶*^, A Judge^1,2^, R N Batra^1^, A Price^1^, A D Liddle^1^, M K Javaid^1,2^, C Cooper^1,2^, D Murray^1^, N K Arden^1,2^.

^1^ Musculoskeletal Epidemiology. Nuffield Department of Orthopaedics, Rheumatology and Musculoskeletal Sciences, University of Oxford, Oxford, UK

^2^ MRC Lifecourse Epidemiology Unit, University of Southampton, Southampton General Hospital, Southampton, UK

^3^ Arthritis research UK Centre for Sport, Exercise and Osteoarthritis. Nuffield Department of Orthopaedics, Rheumatology and Musculoskeletal Sciences, University of Oxford, Oxford, UK

^¶^ These authors contributed equally to this work.

*Corresponding Author: Cesar Garriga

Nuffield Department of Orthopaedics, Rheumatology and Musculoskeletal Sciences,

University of Oxford,

Windmill Road, Headington,

Oxford, OX3 7LD, UK.

Email: [cesar.garriga-fuentes@ndorms.ox.ac.uk](mailto:cesar.garriga-fuentes@ndorms.ox.ac.uk)

Alternate corresponding author: MT Sanchez-Santos ([maria.sanchez@ndorms.ox.ac.uk](mailto:maria.sanchez@ndorms.ox.ac.uk) )

**Supplementary Table S1.** Patients’ completing or not pre- and post-operative OKS questionnaires for development dataset, and post-operative OKS questionnaires for validation dataset.

| **Variables** | **KAT** | | | **COASt** | | |
| --- | --- | --- | --- | --- | --- | --- |
| ***Outcome*** | **Responders n=1,649** | **Non-responders n=603** | ***P*-value** | **Responders n=595** | **Non-responders n=251** | ***P*-value** |
| OKS at 1 year of follow-up (units, 0 worst – 48 best, median, IQR) | 36 (27-42) | 33 (25-42) | 0.259* | 39 (30-44) | ─ | ─ |
| ***Patients’ characteristics*** |  |  |  |  |  |  |
| Age (years), mean (SD) | 70 (8) | 71 (9) | 0.053 | 70 (9) | 69 (12) | 0.145 |
| Female, n (%) | 921 (55.9%) | 350 (58.0%) | 0.353 | 339 (57.0%) | 148 (59.0%) | 0.593 |
| Marital status, n (%) |  |  |  |  |  |  |
| *Married* | 1,082 (66.0%) | 376 (65.4%) | 0.348 | 330 (67.1%) | 24 (64.9%) | 0.028 |
| *Single* | 65 (4.0%) | 31 (5.4%) |  | 20 (4.1%) | 5 (13.5%) |  |
| *Widowed/Divorced* | 492 (30.0%) | 168 (29.2%) |  | 142 (28.9%) | 8 (21.6%) |  |
| IMD 2004 score, medium (IQR) | 15.6 (9.6-25.5) | 14.5 (8.9-24.9) | 0.273 | 10.2 (6.3-16.9) | 10.6 (6.1-18.8) | 0.238 |
| BMI (kg/m^2^), mean (SD) | 29.7 (5.4) | 29.6 (5.3) | 0.925 | 30.7 (5.5) | 30.9 (6.6) | 0.747 |
| Anxiety/depression (EQ5D-3L 5th question) | 643 (39.3%) | 244 (46.7) | 0.003 | 173 (32.3%) | 35 (47.3%) | 0.011 |
| Pre-operative OKS, mean (SD) | 18.3 (7.5) | 17.0 (7.7) | <0.001 | 19.3 (7.7) | 17.6 (7.5) | 0.058 |
| ***Clinical factors*** |  |  |  |  |  |  |
| ASA grade, n (%) |  |  |  |  |  |  |
| *Fit and healthy* | 277 (17.5%) | 89 (15.8%) | 0.013 | 49 (9.4%) | 4 (12.5%) | 0.398 |
| *Asymptomatic no restriction* | 991 (62.8%) | 329 (58.5%) |  | 381 (73.4%) | 20 (62.5%) |  |
| *Symptomatic minimal/severe restriction* | 311 (19.7%) | 144 (25.6%) |  | 89 (17.2%) | 8 (25.0%) |  |
| Disease Type, n (%) |  |  |  |  |  |  |
| *OA* | 1,561 (95.3%) | 541 (94.6%) | 0.492 | 465 (94.5%) | 31 (93.9%) | 0.889 |
| *RA* | 77 (4.7%) | 31 (5.4%) |  | 27 (5.5%) | 2 (6.1%) |  |
| Disease side, n (%) |  |  |  |  |  |  |
| *One knee* | 432 (26.4%) | 137 (24.0%) | 0.521 | 83 (20.2%) | 10 (18.9%) | 0.012 |
| *Both knees* | 642 (39.2%) | 232 (40.6%) |  | 192 (46.8%) | 15 (28.3%) |  |
| *General* | 564 (34.4%) | 203 (35.5%) |  | 135 (32.9%) | 28 (52.8%) |  |
| Knee arthroscopy, n (%) | 218 (13.3%) | 74 (32.9%) | 0.821 | 56 (10.6%) | 4 (10.3%) | 0.945 |
| Other condition affecting mobility, n (%) | 224 (13.7%) | 94 (16.6%) | 0.096 | 487 (89.7%) | 36 (90.0%) | 0.950 |
| ***Pre-operative Surgical factors*** |  |  |  |  |  |  |
| Fixed flexion deformity, n (%) | 930 (57.4%) | 311 (54.8%) | 0.272 | 254 (62.7%) | 32 (57.1%) | 0.421 |
| Damaged/Absent ACL, n (%) | 558 (34.6%) | 201 (35.7%) | 0.678 | 93 (24.8%) | 9 (17.3%) | 0.235 |

OKS, Oxford knee score; BMI, Body mass index; SF-12, Sort form; IMD, Index of Multiple deprivation; IQR: interquartile range; OA, Osteoarthritis; RA, Rheumatology Arthritis; ASA, American Society of Anaesthesiologists; TKR, Total knee replacement; ACL, anterior cruciate ligament; PCL, posterior cruciate ligament.

The t-tests are used for continuous variables and χ^2^ tests for categorical variables. Where continuous variables were not normally distributed, a non-parametric t-test (Kruskal-Wallis) was used.

* Wilcoxon test.

**Supplementary Table S2**. Number (percent) of patients with missing data for the overall and divided by gender

| **Characteristic, n (%)** | **KAT (n=1,649)** | **COASt (n=595)** |
| --- | --- | --- |
| Age (years) | - | - |
| Marital status | 10 (0.6) | 103 (17.3) |
| IMD 2004 score | 623 (37.8) | 1 (0.2) |
| BMI (kg/m^2^) | 52 (3.2) | - |
| Pre-operative OKS | - | 53 (8.9) |
| EQ5D-3L 5^th^ question | 13 (0.8) | 60 (10.1) |
| ASA grade | 70 (4.2) | 76 (12.8) |
| Disease Type | 11 (0.7) | 103 (17.3) |
| Disease side | 11 (0.7) | 185 (31.1) |
| Previous knee arthroscopy | 11 (0.7) | 67 (11.3) |
| Other condition affecting mobility | 14 (0.9) | 52 (8.7) |
| Fixed flexion deformity | 29 (1.8) | 190 (31.9) |
| Pre-operative ACL | 37 (2.2) | 220 (36.9) |

KAT, Knee Arthroplasty Trial; COASt, clinical outcomes in arthroplasty study; TKR, Total knee replacement; OKS, Oxford knee score; mv, missing values; sd, standard deviation; BMI, body mass index; EQ-5D, EuroQol five dimensions questionnaire; IMD, Index of Multiple Deprivation; ASA, Physical Status Classification System of the American Society of Anaesthesiologists; ACL, anterior cruciate ligament.
